# Active learning for data efficient semantic segmentation of canine bones in radiographs

**DOI:** 10.3389/frai.2022.939967

**Published:** 2022-10-26

**Authors:** D. E. Moreira da Silva, Lio Gonçalves, Pedro Franco-Gonçalo, Bruno Colaço, Sofia Alves-Pimenta, Mário Ginja, Manuel Ferreira, Vitor Filipe

**Affiliations:** ^1^School of Science and Technology, University of Trás-os-Montes e Alto Douro (UTAD), Vila Real, Portugal; ^2^INESC Technology and Science (INESC TEC), Porto, Portugal; ^3^Department of Veterinary Science, UTAD, Vila Real, Portugal; ^4^Veterinary and Animal Research Centre (CECAV), Vila Real, Portugal; ^5^Department of Animal Science, UTAD, Vila Real, Portugal; ^6^Centre for the Research and Technology of Agro-Environmental and Biological Sciences (CITAB), Vila Real, Portugal; ^7^Neadvance Machine Vision SA, Braga, Portugal

**Keywords:** X-ray image analysis, deep learning, active learning, cluster based sampling, representative sampling, Monte Carlo Dropout sampling, Shannon's entropy

## Abstract

X-ray bone semantic segmentation is one crucial task in medical imaging. Due to deep learning's emergence, it was possible to build high-precision models. However, these models require a large quantity of annotated data. Furthermore, semantic segmentation requires pixel-wise labeling, thus being a highly time-consuming task. In the case of hip joints, there is still a need for increased anatomic knowledge due to the intrinsic nature of the femur and acetabulum. Active learning aims to maximize the model's performance with the least possible amount of data. In this work, we propose and compare the use of different queries, including uncertainty and diversity-based queries. Our results show that the proposed methods permit state-of-the-art performance using only 81.02% of the data, with O(1) time complexity.

## 1. Introduction

Image segmentation is one of the most important yet, challenging tasks in medical image analysis (Shah and Sharma, 2018). In recent years, Deep Learning's (DL) emergence has made it possible to build models that achieve human-like or superior performance in many medical imaging tasks, such as segmentation. However, the DL shortcoming is the need for large quantities of annotated data, often in the order of thousands (Ronneberger et al., 2015). Additionally, gathering such large and high-quality datasets, annotated by medical experts, is often very difficult (Kim et al., 2019; Nguyen et al., 2021) because segmentation requires thorough pixel-wise labeling, hence being a highly time-intensive procedure (Ozdemir et al., 2021). Additionally, collecting medical images might be financially expensive (Kim et al., 2019). Active Learning's (AL) goal is to identify which unlabeled samples are the most interesting to be labeled by a human expert. In other words, how to maximize the model's performance using the least possible amount of data (Ren et al., 2020). Without AL, this sampling is purely random, which might cause data redundancy. Hence, the use of AL enables the annotation process to be as time and financially efficient as possible.

In this work, we explore the use of AL methodologies in the context of the segmentation of dogs' femur and acetabulum bones in X-ray images. Dogs' coxofemoral joint radiographic examinations are used worldwide for screening hip dysplasia and to select better animals for breeding. Radiographic evaluation is performed by human observation, being considered a time-consuming, expensive and relatively subjective process due to differences in classification between evaluators. Precise segmentation of these bone structures is noteworthy as it allows further automated diagnosis of canine hip dysplasia (Moreira da Silva et al., 2021, 2022). However, the joint regions present high noise, low contrast, overlapping tissue, and a narrow gap between the femur and acetabulum (Lianghui et al., 2019). As such, the annotation of these regions requires increased attention, a greater level of medical specialization and knowledge of these anatomical structures. Consequently, this set of factors leads to an increased expenditure of veterinary medicine professionals' valuable time in the annotation process. Therefore, we aim to assemble and compare the effects of different AL queries to build a high performant U-Net model with low amounts of annotated data. The developed techniques will be integrated into the Dys4Vet^1^ web platform, a dedicated software for the automated canine hip dysplasia diagnosis.

The rest of this paper is organized as follows: Related work (Section 2); Methods (Section 3); Results and discussion (Section 4); Conclusions (Section 5).

## 2. Related work

There are two types of AL queries (Munro, 2021): uncertainty sampling; diversity sampling. The first aims to fix the model's currently known confusion by sampling data that the model presents low predictive confidence. Diversity sampling intends to provide the model with samples of unknown areas of the feature space, thus narrowing the model's knowledge gap. Mahapatra et al. (2018) used AL for X-Ray lung segmentation. The authors suggest generative adversarial networks (GANs) to generate diverse images. Then, using a Bayesian Neural Network, each generated sample's informativeness is calculated. The informativeness is calculated through the combination of epistemic and aleatoric uncertainties (Kendall and Gal, 2017). The most informative samples are added, at each iteration, to the labeled training data. With this method, the authors reach state-of-the-art performance by using only 35% of the full annotated dataset. Ozdemir et al. (2018) used Monte Carlo Dropout (Gal and Ghahramani, 2016) (MCD) to measure sample uncertainty based on inference variance. Then, content distance (Gatys et al., 2016) and layer entropy maximization are used to measure representativeness. The novelty of this work is that instead of applying uncertainty and then sampling for diversity, the authors propose a Borda count approach: samples are ranked for each metric, and sampling is carried out based on combined rank. On a similar note, in later works, Ozdemir et al. (2021) proposed a modification of MCD (Gal and Ghahramani, 2016), where instead of randomly dropping neuron connections, entire convolutional kernels are dropped. The uncertainty of each sample is calculated by averaging each pixel's variance over the multiple inferences. Additionally, a variational autoencoder is used to project gaussian distributions of labeled and unlabeled pools. With both distributions, the authors calculate underrepresented samples in the labeled dataset. By combining uncertainty and representativeness, the authors stay within 2% of the state-of-the-art performance using only 25% of the data. Zhao et al. (2020) modified a U-Net to extract and then upscale segmentation maps from deep and intermediate layers. Then, the authors calculate the dice coefficient between the model's final segmentation map and each upscaled map. The samples with the highest dice scores' average are selected to be labeled. This technique achieves comparable state-of-the-art performance with 43.16% of the data. However, the results do not differ much from random sampling, with a performance difference of <1%. Zhao et al. (2021) introduced DSAL through the reuse of the previously described technique. While high uncertainty samples are annotated by a human expert, in this work the samples with low uncertainty are also provided to weak labelers (i.e., dense conditional random fields) to generate pseudo labels. The authors state the incorporation of pseudo labels further boosts the results. Jin et al. (2022) proposed a one-shot active learning framework based on contrastive learning and diversity sampling. First, contrastive learning is used for feature extraction. Then, this new feature space is clustered using K-Means++ (Arthur and Vassilvitskii, 2007), and sampling is performed using farthest point sampling (FPS). While clustering guarantees inter-cluster diversity, FPS provides intra-cluster diversity. This method was validated in three different datasets, and it delivered dice score gains when compared to others.

## 3. Methods

This section describes the employed methodologies for the present study. Initially, the used dataset is presented in Section 3.1. Then, Section 3.2 describes the DL segmentation model used for the experiments. Lastly, Section 3.3 details the AL procedure and Section 3.4 details the proposed AL queries.

### 3.1. Dataset

**Figure 1:**
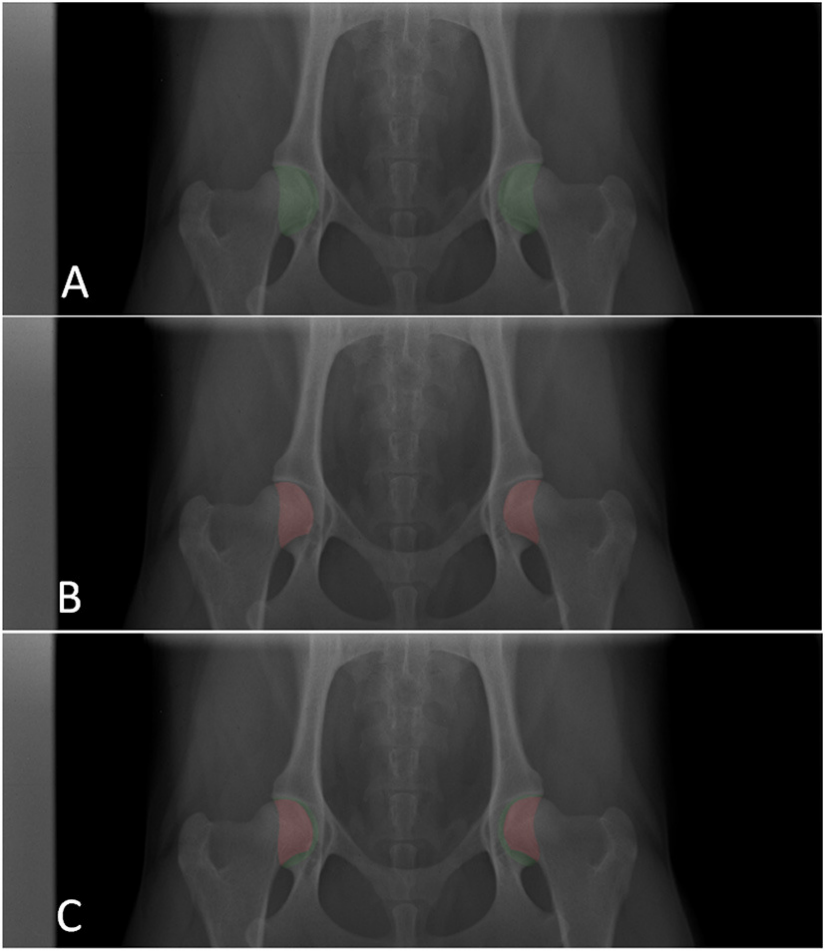
Example of annotations made for each image. **(A)** Acetabulum annotation. **(B)** Femoral head (that overlaps the acetabulum) annotation. **(C)** Combined femoral head and acetabulum annotations. The X-ray was cut on the y axis for better visualization. For model training, full images are used.

For this work, DICOM images were collected from Veterinary Teaching Hospital of the University of Trás-os-Montes and Alto Douro and from the Danish Kennel Club, totaling 202 images. Please note that each image corresponds to a unique patient, avoiding data correlation in subsequent splitting processes. Then, manual annotation was carried out for every DICOM. In detail, the acetabulum and femoral head acetabulum intersection were annotated (Figure 1). With these annotations, three-channel masks were generated, where each channel is a binary mask for each class: background, femur, and acetabulum. Then the images were resized to 544 × 448. The masks underwent the same resizing through nearest neighbor interpolation. Finally, a test (15%) and a validation set (15%) are created, which remain constant throughout the AL cycles. Additionally, 3% is used as initial training data $L_{0}$ and the remaining as the initial unlabeled pool $U_{0}$.

### 3.2. Segmentation model

The DL segmentation model we use is the same we propose in previous works (Moreira da Silva et al., 2022), a U-Net with EfficientNet (Tan and Le, 2019) modules as the feature-extractor backbone.

For quantitative results we evaluated the dice score (Equation 1), as it is the common metric in medical image segmentation (Siddique et al., 2021).


(1) $$\begin{array}{l} Dice\left(P,G\right)=\frac{2\times |G\cap P|}{\left|G|+|P\right|} \end{array}$$

where

P: Predicted Segmentation

G : Ground Truth

We also use the same loss function of the previous work (Moreira da Silva et al., 2022), a combination of dice and focal loss (Equation 2). For this work, we set α = 0.25 and γ = 2.


(2) $$\begin{array}{l} L\left(P,G\right)=\left(1-Dice\left(P,G\right)\right)-\alpha {\left(1-P\right)}^{\gamma }log\left(P\right) \end{array}$$

### 3.3. Active learning procedure

In this section, we present and explain our AL procedures. We define the unlabeled pool as U={X,Y}, where X is the available feature space to sample from, and Y the corresponding labels. Please note that we have Y because we generate the unlabeled pool artificially, as detailed in Section 3.1. In real-world AL, Y would not be present, so the labels would need to be provided by an oracle (i.e., human expert) in real-time.

Our AL cycle is formally defined as follows: Given the initial training data $L_{0}$, and the initial unlabeled pool $U_{0}$, at each AL iteration *t*, a given acquisition query *Q* will sample *n* images from $U_{t}$ and then this new subset $U_{t}^{n}\subseteq U_{t}$ is added to the training dataset $L_{t}=L_{t-1}\;\cup \;U_{t}^{n}$, removed from the next iteration's unlabeled pool $U_{t+1}=U_{t}\backslash U_{t}^{n}$, and the model is re-trained. After training, the model is evaluated on the test dataset, and the resulting metrics are saved. The iteration is incremented *t* = *t* + 1, and this process repeats until the unlabeled pool is empty U=∅. For each query Q under study, this entire cycle is repeated 10 times. Later, we will provide each metric's average at each AL iteration *t*, for each query Q. This way our results are more statistically significant.

### 3.4. Active learning queries

For this study, we employ and compare five different queries:

#### 3.4.1. Random sampling

Randomly sampling *n* elements from the unlabeled pool $U^{n}\in_{R}U$. This query serves as a baseline.

#### 3.4.2. Cluster based sampling

First, the unlabeled pool U is normalized according to (3):


(3) $$\begin{array}{l} U_{N}=\left\{\frac{x-U_{min}}{U_{max}-U_{min}}:x\in U\right\} \end{array}$$

Then the dimensionality of $U_{N}$ is reduced by applying Principal Component Analysis (PCA) (Pearson, 1901), with 99% explained data variance. We define the reduced pool as $U_{R}$. Then, we use K-Means++ (Arthur and Vassilvitskii, 2007) to create *n* clusters inside $U_{R}$. Then, for each centroid of $U_{ℛ}$, the closest element is found. The corresponding *n* elements in U are sampled. Thus, this query ensures maximum diversity between the samples.

#### 3.4.3. Representative sampling

This query requires both the unlabeled pool U and the training data L. We then apply the same two initial steps of the previous query to both U and L. Then, also using K-Means++, two clusters C are created: a training cluster $C_{L}$, and an unlabeled cluster $C_{U}$. For each item in $C_{U}$ it's representativeness R is calculated according to (4):


(4) $$\begin{array}{l} R=\left\{d\left(x,\mu_{C_{L}}\right)-d\left(x,\mu_{C_{U}}\right):x\in C_{U}\right\} \end{array}$$

where

*μ*: Cluster centroid

d: Euclidean distance

R measures the difference between the training and the unlabeled data. The first *n* elements of U that have the highest corresponding R values are sampled. In short, we select the items that best represent the unlabeled pool population and look the most different from the current training data.

#### 3.4.4. Monte Carlo Dropout sampling

Monte Carlo Dropout (MCD) is a Bayesian ANN approximation of the Gaussian process, introduced by Gal and Ghahramani (2016). It uses the dropout layers (Srivastava et al., 2014) of a DL model to measure its predictive uncertainty. It works by turning on dropout during inference, and by running inference *k* times, each dropout configuration corresponds to a Monte Carlo sample from the available models' space. Thus, we obtain a predictive distribution enabling the inspection of the predictive uncertainty. For this study we set *k* = 30. Then we obtain each sample's average prediction $\bar{P}$ according to (5):


(5) $$\begin{array}{l} \bar{P}=\left\{\frac{1}{k}\overset{k}{\underset{i=1}{\sum }}г\left(x\right):x\in U\right\} \end{array}$$

where

г: DL model

Afterwards, each sample's uncertainty is calculated using Shannon's entropy (Shannon, 1948), according to Equation (6). Since the used model uses a sigmoid activation function, we calculate the entropy for the femur (output channel 1) and the acetabulum (output channel 2) class separately, averaging them thereafter.


(6) $$\begin{array}{l} ℰ=\{\frac{1}{2}\sum_{c=1}^{2}\left[\frac{1}{H\times W}\sum_{h=0}^{H}\sum_{w=0}^{W}\left(-x_{hwc}\;log_{2}\;x_{hwc}\right)\right]:x\in \bar{P}\} \end{array}$$

The first *n* elements of U that have the highest corresponding E values are sampled. As such, we select the items that the model presents a higher level of uncertainty. We call this method CWE-MCD (Class-wise Entropy Monte Carlo Dropout).

#### 3.4.5. Representative CWE-MCD

All the previously mentioned queries sample from one of the following perspectives: uncertainty or diversity. This method proposes a combination of two techniques: Representative Sampling and CWE-MCD. To combine both queries, we adopt the Ozdemir et al. (2018)'s Borda count approach. In short, we separately calculate the R and E scores for each image in U. Then each unlabeled image is ranked based on how high each score is. The final sampling is based on the 15 highest combined rank.

## 4. Results and discussion

Initially (*t* = 0), the labeled dataset $L_{0}$ has six train images and the unlabeled pool $U_{0}$ has 131 images, and at each active learning iteration, a given query Q will select 15 images (*n* = 15). We repeat this procedure until we run out of images, resulting in nine AL iterations $\left(t_{max}=\left\lceil \frac{131}{15}\right\rceil =9\right)$. Regarding the model, at each AL iteration, we train a model from scratch, using the Adam optimizer with a learning rate of 1*e* − 2 and a batch size of eight. Additionally, we use two callbacks that monitor the validation's data dice score. The first is a callback that reduces the learning rate by a factor of 0.1 if the metric does not improve after eight epochs. Secondly, a callback that halts the training if the metric does not improve by ten epochs (early stopping). Therefore, we set the number of epochs to 500.

**Figure 2:**
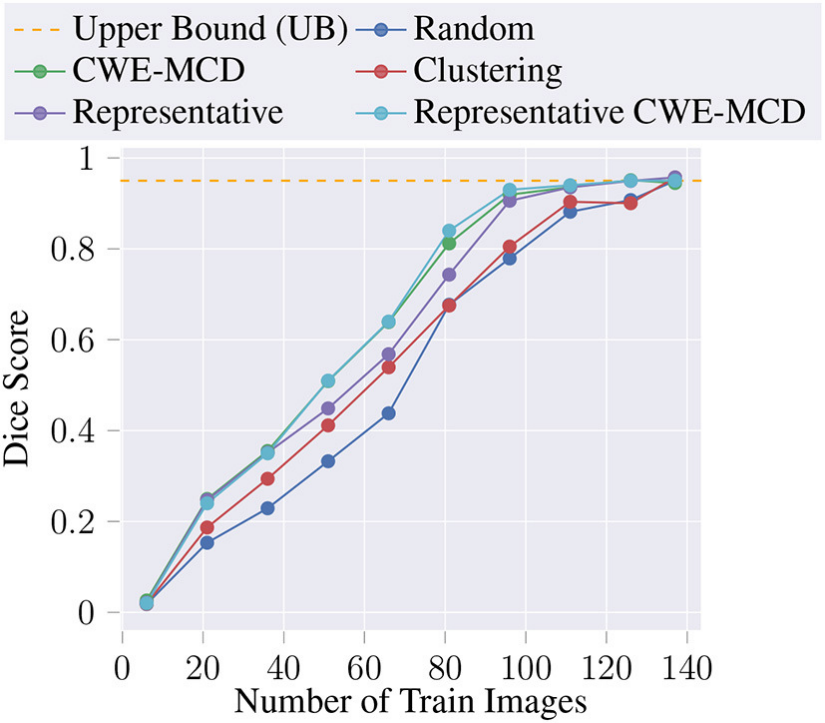
Average dice score at each AL iteration for the proposed methods.

Also, we train the U-Net with the entire unlabeled pool combined with the initial training data ($L_{0}\;\cup \;U$), achieving a test dice score of 0.95. We denote this value as the model's Upper Bound (UB). The average dice score, for each AL iteration, for the proposed methods are described in Table 1, and illustrated in Figure 2. Noticeably, every query (Clustering, Representative, CWE-MCD, Representative CWE-MCD) performed better than the baseline (Random) in all iterations. Despite clustering being a simple technique, in the first iterations, it outperforms the baseline. As the size and diversity of the unlabeled pool decreases, the performance of the clustering query becomes identical to the baseline. Both the baseline and the clustering queries need the entire unlabeled pool as training data to reach the UB. Nonetheless, clustering still proves suitable in early AL iterations. The three more advanced methods we build (Representative, CWE-MCD, Representative CWE-MCD) show significant dice score gains compared to the baseline. In the first two iterations, these present closely the same performance. From the second, CWE-MCD and Representative CWE-MCD provide superior performance. This fact can corroborate (Ozdemir et al., 2021) statement that uncertainty may not be a sufficiently calibrated metric until the training data size is adequately large. Additionally, Representative CWE-MCD provides slightly better results over CWE-MCD and Representative until the sixth iteration. This is expected, as Representative CWE-MCD combines the best aspects of the diversity and uncertainty sampling, thus being a more powerful technique (Munro, 2021). Despite this slight performance superiority, these three queries can reach the UB at around 111 training images compared to the required 137 when using the baseline or the clustering methods. This means that using these queries allowed the same level of performance with ≈ 81.02% of the data, a saving of ≈ 18.98% (26 images).

**Table 1:** Average dice score at each AL iteration for the proposed methods.

**Query Q**	**AL iteration** ***t***									

	**0**	**1**	**2**	**3**	**4**	**5**	**6**	**7**	**8**	**9**
Random	0.02	0.15	0.24	0.34	0.47	0.66	0.79	0.89	0.91	0.95
Clustering	0.02	0.19	0.30	0.39	0.51	0.68	0.81	0.90	0.91	0.95
Representative	0.02	0.25	0.36	0.45	0.58	0.75	0.89	0.94	0.95	0.95
CWE-MCD	0.02	0.23	0.34	0.49	0.63	0.82	0.92	0.94	0.95	0.95
Representative CWE-MCD	0.02	0.24	0.35	0.51	0.64	0.84	0.93	0.94	0.95	0.95

**Figure 3:**
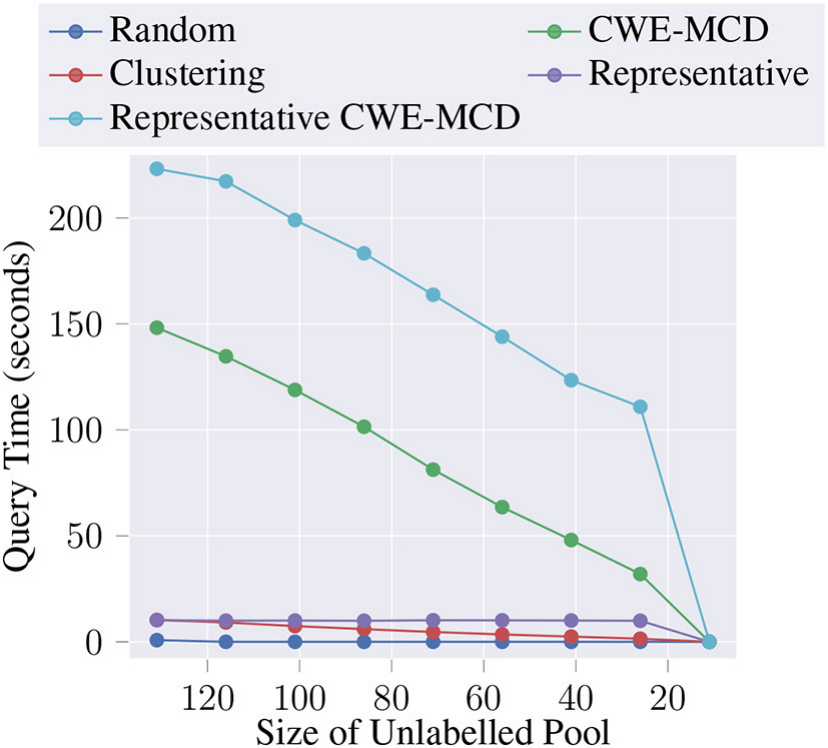
Average query time (in seconds) at each AL iteration for the proposed methods.

Additionally, we decided to measure the average query time in seconds, at each AL iteration, for each of the proposed queries. Figure 3 depicts the results. As expected, the baseline is almost instant. The clustering technique is also pretty fast, decreasing times as the iterations increase. One of the best performing queries, CWE-MCD, presents a significantly higher time complexity O(n), meaning that the required time scales linearly to the size of the unlabeled pool. In practice, this query might be unsuitable for large unlabeled pools. Thus, to offset the computation times, it might be required to tune the number of Monte Carlo samples (i.e., number of inferences). Notwithstanding, representative query presents a linear time complexity O(1) while still delivering noteworthy dice score gains, thus being suitable for large unlabeled pools. Lastly, Representative CWE-MCD presents the same behavior as CWE-MCD but with a significant additional time overhead due to representativeness and Borda count computations. In practice, the use of this query will be even more limited than the CWE-MCD due to the increased times. However, its use can still be advantageous in situations that demand maximum performance, situations where time is not a constraint, or when applied to a small subset of the unlabeled pool.

## 5. Conclusions

In this work, we study and compare the effectiveness of different AL query strategies in the ambit of the segmentation of dogs' femur and acetabulum bones in X-ray images. In detail, we suggest measuring the uncertainty by calculating class-wise entropy using Monte Carlo Dropout. Furthermore, we propose to combine this uncertainty metric with a representativeness method, inspired by the works of Ozdemir et al. (2018). This method is superior to the others, allowing an 18.98% reduction in the amount of required annotated data. Despite this method being O(n) in time complexity, representative sampling is O(1) time complex, with comparable performance levels, thus suitable for large unlabeled datasets. For future research, we intend to study other possible combinations of the entropy and representativeness methods presented in this paper. In addition, the creation of annotation software unified with an AL framework, equipped with automatic pre-annotation capabilities, would allow further time savings for veterinary professionals.

## Data availability statement

The raw data supporting the conclusions of this article will be made available by the authors, without undue reservation.

## Author contributions

DM, LG, and VF contributed to conception and design of the study. PF-G, BC, SA-P, and MF organized the database. DM, LG, MF, and VF defined the methodology. DM, LG, MG, and VF performed validation and data analysis. DM wrote the first draft of the manuscript. All authors contributed to manuscript revision, read, and approved the submitted version.

## Funding

This work was financed by project Dys4Vet (POCI-01-0247-FEDER-046914), co-financed by the European Regional Development Fund (ERDF) through COMPETE2020—the Operational Programme for Competitiveness and Internationalization (OPCI). The authors are also grateful for all the conditions made available by FCT—Portuguese Foundation for Science and Technology, under the projects UIDB/04033/2020, UIDB/CVT/00772/2020, and Scientific Employment Stimulus—Institutional Call—CEECINST/00127/2018 UTAD.

## Conflict of interest

Author MF was employed by Neadvance Machine Vision SA. The remaining authors declare that the research was conducted in the absence of any commercial or financial relationships that could be construed as a potential conflict of interest.

## Publisher's note

All claims expressed in this article are solely those of the authors and do not necessarily represent those of their affiliated organizations, or those of the publisher, the editors and the reviewers. Any product that may be evaluated in this article, or claim that may be made by its manufacturer, is not guaranteed or endorsed by the publisher.

## Footnotes

^1^ https://www.citab.utad.pt/projects/780/show
